# The effect of extremely low frequency electromagnetic fields following on upregulation of miR-21 and miR-29 in gastric cancer cell line 

**Published:** 2021

**Authors:** Elham Siasi, Elaheh Moniri

**Affiliations:** *Department of Genetic, Collage of Sciences, North Tehran Branch, Islamic Azad University, Tehran, Iran.*

**Keywords:** Gastric cancer cell line, Electromagnetic field, miR-21, miR-29, expression

## Abstract

**Aim:**

Extremely low frequency electromagnetic fields affect miRNAs expression in cancer cell. In this study, electromagnetic fields exposed to low frequency were used to compare miR-21 and miR-29 expressions in a gastric cancer cell line.

**Background:**

It has been recently suggested that the low frequency electromagnetic fields probably function as a treatment for cancers.

**Methods:**

A cultured cell line of gastric cancer was exposed to an electromagnetic radiation system. The cell line was assigned to 4 groups under continuous and discontinuous radiations of 0.25 and 2.5 ml Tesla field strength. Then, the groups were compared with a non-radiation control group. Later, RNA extraction and cDNA synthesis were prepared for miR-21 and miR-29. Real Time PCR method was used to determine how expressions of these two microRNAs differ. Finally, the results were statistically analyzed.

**Results:**

The percentage of cell viability in the electromagnetic field radiation experienced a significant decrease compared to that of the control group. In addition, expression of miRNA-21 and miRNA-29 had a significant increase as the strength of the electromagnetic field radiations was on an upward trend. Similarly, the percentage of cell viability saw a significant decline in the upregulation of miRNA-21 and miRNA-29 regardless of radiation types.

**Conclusion:**

Findings of this study showed the therapeutic effect of low frequency electromagnetic fields on the gastric cancer cell line. They also indicated that novel biomarkers (miRNA-21 and miRNA-29) could be proposed as potential treatments of gastric cancer, but the results are required to be well established by future studies.

## Introduction

 Cancer is a stage of disease in which a group of cells grows in an uncontrollable manner (1-4). Gastric cancer has been always considered as a widespread lethal disease particularly among the elderly male. Stomach cancer is the fifth prevalent neoplasm, holding the third position in the ranking of fatal cancers (1, 2). As a disease is involved with a myriad of factors, the gastric cancer is closely associated with its risk factors namely genetic factors (mutation in P53, MCC, and APC), epigenetic factors (dysregulation of gene expression in GC), and environmental factors (*Helicobacter pylori* infection, gastric ulcer, gastroesophageal reflux disease, alcohol, smoking, obesity, radiation, Epstein Barr virus infection, blood group, socioeconomic status, and gender (the male twice in number) (1-4). However, Western countries are reported with fewer cases of gastric cancer than the Asians like Iran, where this type of cancer is reported as the deadliest nationwide (4, 5). The treatment of cancer involves insistent methods such as surgery, radiation therapy, and chemotherapy (6). Electromagnetic fields are used as beneficial tools in medical diagnosis. Interactions between electromagnetic fields and living matter cause effects such as initiating, accelerating, or inhibiting biological processes (7). Although treatment methods of cancer such as chemotherapy and radiation therapy may kill cancer cells by inducing apoptosis, these cells mostly become resistant to such types of therapies. Furthermore, many cancer therapies indirectly turn on apoptosis by chemical or physical damage to DNA (6, 7). The studies, relying on cancer cell culture and animal tumor models, demonstrate that electromagnetic therapy (EMT) terminates the tumor cell and it may have cytotoxic effects on cancer (6, 7). As suggested by a large number of studies, an exposure to electromagnetic fields of very low frequencies (ELF-EMF) may make a wide range of biological effects (6, 7). According to the latest studies, being occupationally exposed to ELF-EMF is a risk factor by itself since it is accompanied by not only a rise in oxidative stress in antibody secretion as well as nitric oxide levels, but also a reduction in melatonin production. These were represented as possible mechanisms responsible for the effects of ELF-EMF on cancer pathogenesis (8-10). Many studies have investigated the anti-tumor effects of magnetic fields (11-16). Extremely Low Frequency Magnetic Fields (ELF-EMFs) have been found able to induce biological changes namely improving the immune function, regulating the oncogenic or tumor suppressive gene expressions, and inhibiting cancer cell proliferation (6, 7). It is worth mentioning that safety is an advantage of ELF-EMFs over chemotherapy and radiotherapy. In a study concerning human toxicity, patients with advanced cancer treated with ELF-EMFs showed no toxicity and other adverse side effects (10-16). However, the detailed mechanisms behind the anti-tumor effects of ELF-EMFs remain unknown, and this in turn limits the clinical application of ELF-EMFs treatment (6, 7). Additionally, there have been investigations on the interaction between effects of ELF-EMFs and microRNA expression (17-20). MiRNA expression profile was altered differentially in the cancer cell after experimental exposure to electromagnetic field and could be used for cancer therapy (19, 20). MicroRNA, with 20-24 nucleotides, is a short class of non-coding RNA. It is able to aim at 3'-untranslated regions (3'-UTRs) of mRNA and is responsible for regulating its expression by either degradation of an mRNA or suppression of its translation (5, 21, 25). Furthermore, one kind of miRNA can target several kinds of mRNAs at a post-transcriptional level, and various miRNAs are able to target identical genes (22-26). Moreover, microRNA plays a key role in proliferation and progression of tumor cells, this not only mediates the cells growth, invasion, migration and apoptosis, but also reinforces anticancer medications (27-30). Therefore, different miRNAs can be considered as prognostic biomarkers in a wide range of human cancers namely gastric cancer (5, 21-24). Previous studies demonstrated that miR 21 and miR 29 play important roles in processes signaling the gastric cancer related cells (5, 21-32). The expression of miR-21 is elevated in both gastric cancer tissues and gastric cancer derived cell lines (5, 21-27). MiR-21 has been demonstrated to accelerate tumorigenesis by targeting tumor suppressor genes, including *phosphatase* and *tensin homolog (PTEN), tropomyosin 1 (TPM1*) and *programmed cell death 4 (PDCD4).* This, in turn, increases tumor cell growth, migration and invasion (21, 22, 27-26). Meanwhile, a variety of cancer types have been reported to suffer from an absence of miR-29 regulation, which predominantly functions as a tumor suppressor (28, 32). When compared to the normal tissues in cell lines or the ones found in animal models, the tissues infected by gastric cancer undergo a significant downregulation of the miR-29 expression. *ITGB1 (integrin β1)* is a relatively new downstream target gene of miR-29 and has a pivotal role in cell signaling, differentiation, migration, and apoptosis of the processes required for gastric carcinogenesis to be evolved and developed (27-32). Thus, we hypothesized that miRNAs upregulated by ELF-EMFs exposure (10 Hz, 0.25 and 2.5 ml T) might influence gastric cancer risk with continuous and discontinuous radiations. So far, there have been no reports on the miRNA expression profile following induction exposed to ELF-EMFs in a gastric cancer cell model. The main objective of this study was to investigate the miRNAs expression of the gastric cancer cell line following an exposure to ELF-EMFs. Then, the differentially expressed miRNAs might be potential therapeutic targets for gastric cancer. Considering the biological role of microRNA, we provided a new context to examine if the ELF-EMFs exposure can be taken as an anti-gastric cancer activity or support tumor progression. 

## Methods

**Study design**

The study was approved by Islamic Azad University Ethics Committee and the authors followed the regulations of the World’s Association Declaration of Helsinki. It was carried out from January 2019 to January 2020 and methods were in line with the required guidelines.

**Cell culture**

According to the previously published studies using one cancer cell culture (33), we selected Human Gastric Cancer Cells (AGS), obtained from the Iran Bank of Genetic Cell Cultures (Cell No. C10071) (Iranian Resource Biological Center). The cells were preserved in EMEM (biowest), containing 10% calf serum (Gibco), 100 units/ml penicillin-streptomycin (amgis), 25% Trypsin with EDTA (Invitrogen), 1% sodium pyruvate and 2 mM Glutamine (Gibco). Under a humidified atmosphere with 5% CO2 at 37C. The cells were kept on the exponential growth as monolayers in plastic tissue-culture flasks. 

**Exposure procedure**

As clarified earlier, the Foundation had already designed and provided the exposure system to simply test the exposures of electromagnetic fields. The exposure system consisted of a power frequency generator, an arbitrary function generator, a narrow band amplifier, and four rectangular waveguides. The setup generated a vertical EMF composed of 1200- windings in 4 coil systems (with 1mm diameter on PVC with 30x12 cm), and placed inside a metal chamber. The system possessed two identical exposure chambers for the continuous and discontinuous exposures in turn. Control and exposure cell dishes were at the same time positioned into an incubator with constant environmental conditions (37°C, 5% CO2). After overnight starvation, ASG cells were exposed to a 10 Hz ELF-EMF at magnetic intensities of 0.25 ml T and 2.5 ml T for 18 h with two types of continuous and discontinuous radiations. The entire magnetic apparatus was located in a hood with humidity and temperature controllers. 

**Evaluation of cell viability**

**Table 1:** Primers for Real Time PCR

gene	Primer 5' – 3'	Reference
miR-21	F- GGGGTAGCTTATCAGACTGATGTT R- GCGAGCACAGAATTAATACGACTC	
miR-29	F- GGTA CCGGTTGTCTTGGGTTTATTG R- GAAT TCAAATACTTCAGAGCTG	
U6	F- CGCAAGGATGACACGCAAATTC R- GCGAGCACAGAATTAATACGACTC	

A cell counting MTT reagent (3-(4, 5-dimethylthiazolyl-2)-2, 5-diphenyltetrazolium bromide)) was used to evaluate cell viability. Briefly, 1000 cells per well were seeded into 96-well plates in 100 μl of cell culture medium, and a 10 Hz ELF-EMFs was later applied at different magnetic intensities for 18 h. In the next stage, the medium was mixed with 10 μl of the reaction solution and incubated for 2 h at 37°C. Then, 100 μL Detergent Reagent was added to the mix and it was left at room temperature in the dark for 2 h. The record absorbance was measured at 570 nm using a microtiter plate reader. Each measurement was repeated 3 times.

**RNA extraction and real time PCR**

According to the manufacturer's instructions, after gastric cancer cells were exposed to a 10 Hz ELF-EMFs, a TRIzol Reagent Kit (Invitrogen) was used to extract the total RNA from the cells. The fluorometric method was applied in order to conduct a quantitative RNA analysis. Total RNA (10 μl) was transcribed into cDNA using a microRNA First-Strand cDNA Synthesis Kit (Pars Genome, Iran). This was carried out according to the manufacturer’s protocol under the reactive condition of 37°C for 60 min, then 95°C for 5 min. MiRNA expression was examined using the Bio-Rad IQ5 Detection System and SYBR Green PCR Master mix (Takara, Japan), as described (for miRNA SYBR Green assay) according to manufacturer's instructions (34). Each Real Time PCR amplification reaction (20 μl total volume) contained 2 μl of cDNA, 10 μl of 2× SYBR1 Green Real Time PCR Master Mix, 0.8 μl of each of the forward, and reverse primer and 6.4 μl of ultrapure water. The samples were heated (at 95°C) for 20 sec so that they could be denatured; subsequently, they underwent 40 cycles of amplification (95°C for 10 sec, 60°C for 20 sec, and 70°C for 6 sec). Table 1 represents the sequences of this study primers as described in previous studies (5, 31). The cycle threshold (Ct) method and the 2-ΔCt formula were in use to normalize expression levels of miRNAs to that of U6 in each sample. Each measurement was repeated three times and normalized against the control group.

**Network analysis**

To more strictly predict target pathways of different expressions of miRNAs in the Target genes, we employed Cytoscape software. It revealed that miRNA processing steps may follow recognized processing routes, and/or many non-recognized miRNA biogenesis pathways, which crosstalk with other cellular pathways (33). 

**Statistical analysis**

The data concerning a minimum of two independent experiments in duplicate were expressed as the mean ± SD. A two-way ANOVA and a t-test were administered to ensure if the differences were significant (if any) between the control and the ELF-EMFs groups (p<0.05).

## Results

**The effects of 10 Hz ELF-EMFs exposures on the viability of gastric cancer cell line**

**Figure 1:**
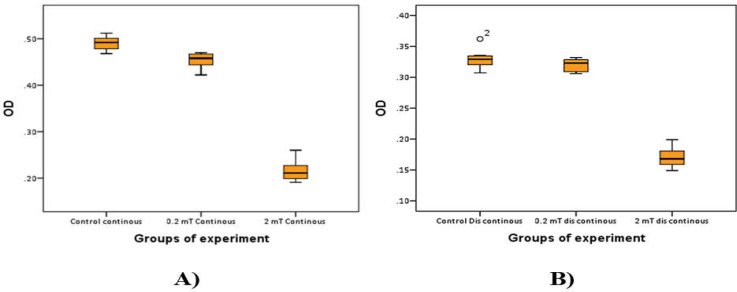
Effects of 10 Hz ELF-EMFs Exposure on the Viability of Gastric Cancer Cell Line (A: Continuous radiation, B: Discontinuous radiation)

In order to explore the impact of the ELF-EMFs on the growth of gastric cancer cells, the cell viability was detected with the MTT assay following a 10 Hz ELF-EMFs exposure at different magnetic intensities for 18 h. MTT assay results showed that 10 Hz ELF-EMFs markedly influence the viability of gastric cancer cells (Figure 1).

**ELF-MFs exposure modulates miR-21 and miR-29 expressions in tumor cells**– 

**Figure 2:**
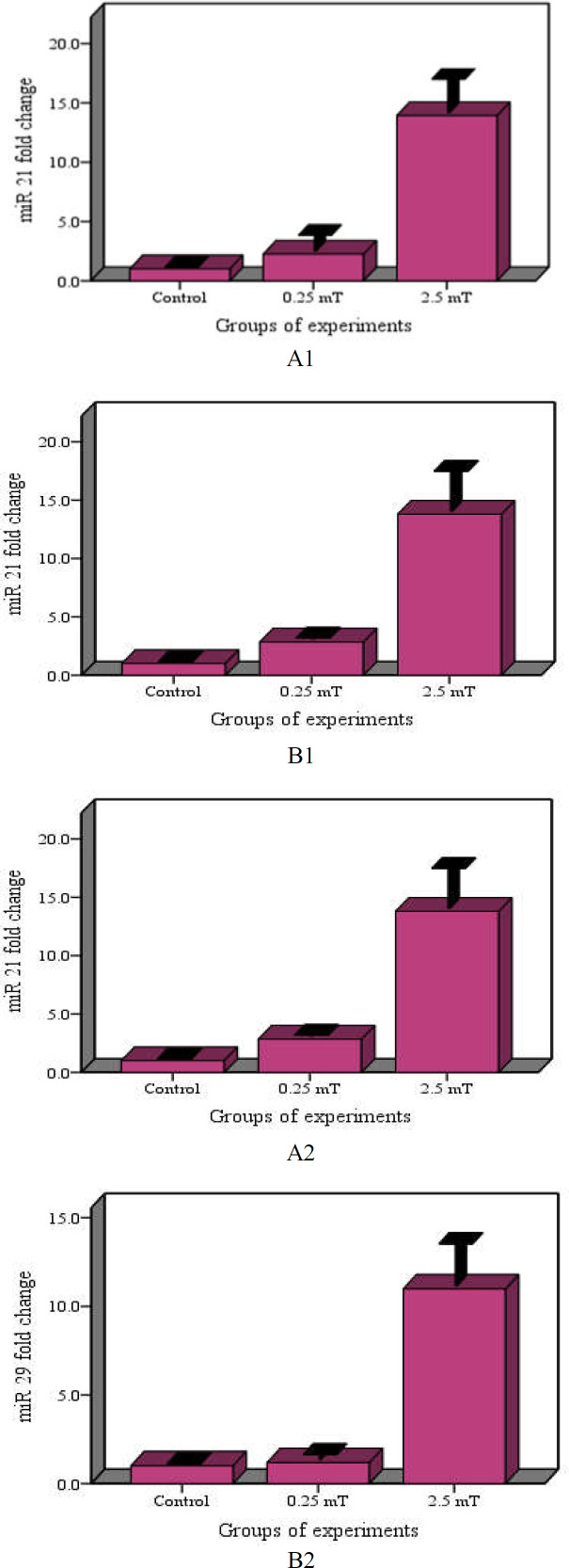
ELF-MFs Exposure changes miR-21 (A) and miR-29 (B) Expression in Tumor Cells. (A1, B1: Continuous radiation and A2, B2: Discontinuous radiation).

Previous studies showed that treatment by ELF-EMFs could modulate the expression of miRNAs (18-20, 27-30). We, therefore, determined the effect of ELF-EMFs on two gastric cancer related miRNAs in a gastric cancer cell line (Figure 2). Two miRNAs (miR-21 and miR-29) were differentially expressed between the LF-MFs and sham groups (Tables 2). It must be noted that miR-29 expression was significantly upregulated in the ELF-EMFs treated tumor cell line. It was also found that the expression of miR-21 in gastric cancer cells with ELF-EMFs treatment (compared to control group) significantly increased after exposure to ELF-MFs.

**Correlation of miR-21 and miR-29 expressions with exposure ELF-MFs in tumor cell line**

**Figure 3:**
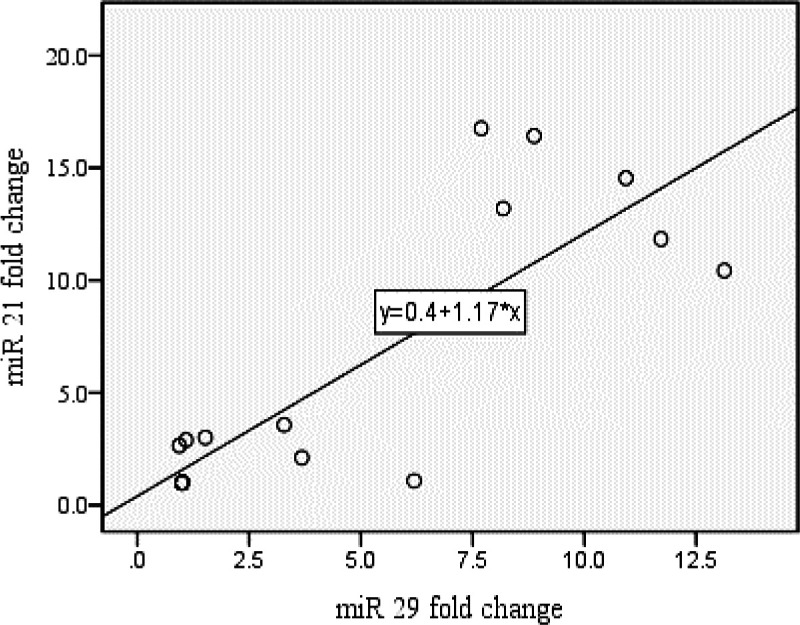
Correlation between the expression of miR-21 and miR-29 levels after exposure to ELF-EMFs. (R=0.807, p value <0.001)

As could be seen in Figure 2, the tumor cell line exposed to ELF-EMFs had higher expressions of miR-21 and miR-29 than those of the control groups. Regression analysis also showed a positive correlation between expression of miR-21 and miR-29 levels after exposure to ELF-EMFs (R=0.807, p value <0.001) (as shown in Figure.3).

**associations between miR-21 and miR-29 expressions and cell viability after being exposed to ELF-EMFs**–

As illustrated by the results of the analysis, there was a perverse relation between miR-21 and miR-29 expression and cell viability after exposure to ELF-EMFs with both types of radiations.

**Figure 4:**
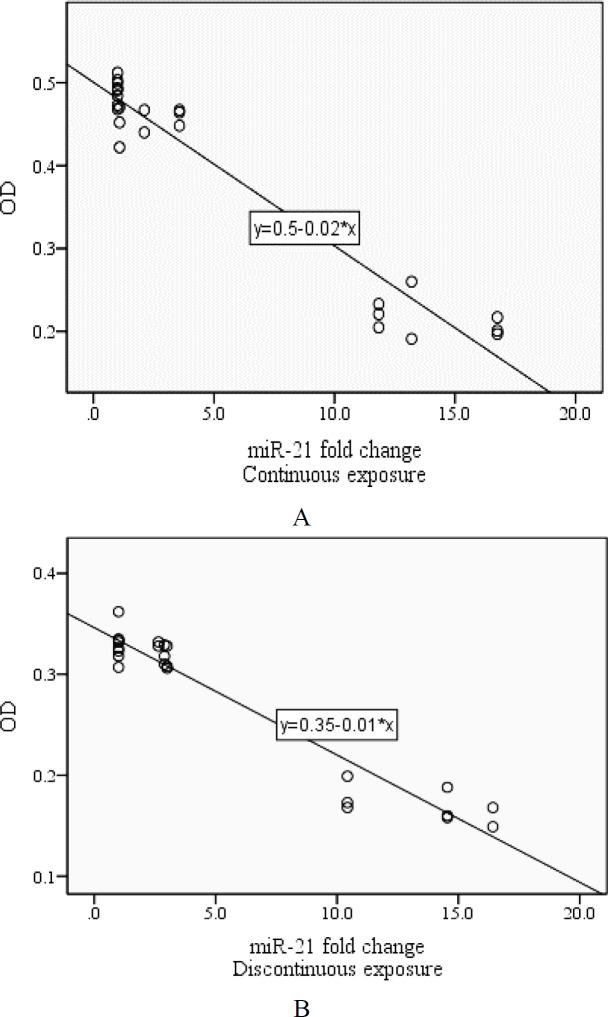
Associations between miR-21 expression and Cell viability after being exposed to ELF-EMFs. (A: Continuous radiation (R= -0.918, p value <0.001), B: Discontinuous radiation (R= -0.889, p value <0.001))

**Figure 5:**
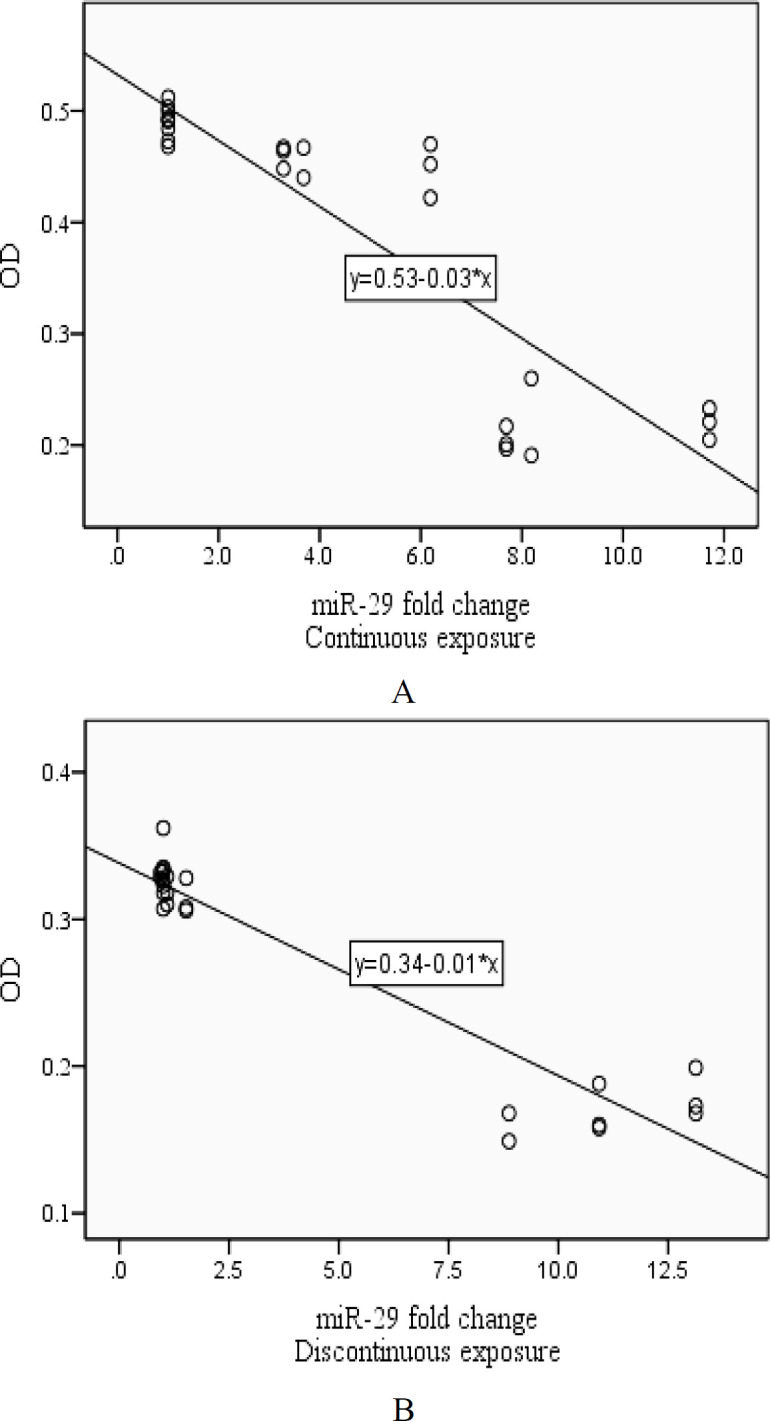
Associations between miR-29 expression and Cell viability after being exposed to ELF-EMFs. (A: Continuous radiation (R= -0.828, p value <0.001), B: Discontinuous radiation (R= -0.800, p value <0.001))

The analysis showed that associations between miR-21 expression and cell viability were R= -0.918 after being exposed to ELF-EMFs (p value <0.001) with continuous radiation and R= -0.889 (p value <0.001) with discontinuous radiation. Besides, following the exposure to ELF-EMFs, associations between miR-29 expression and cell viability were R= -0.828 and R= -0.800 (p value <0.001) with continuous and discontinuous radiation respectively (Figures 4 and 5).

**Network analysis**

**Figure 6:**
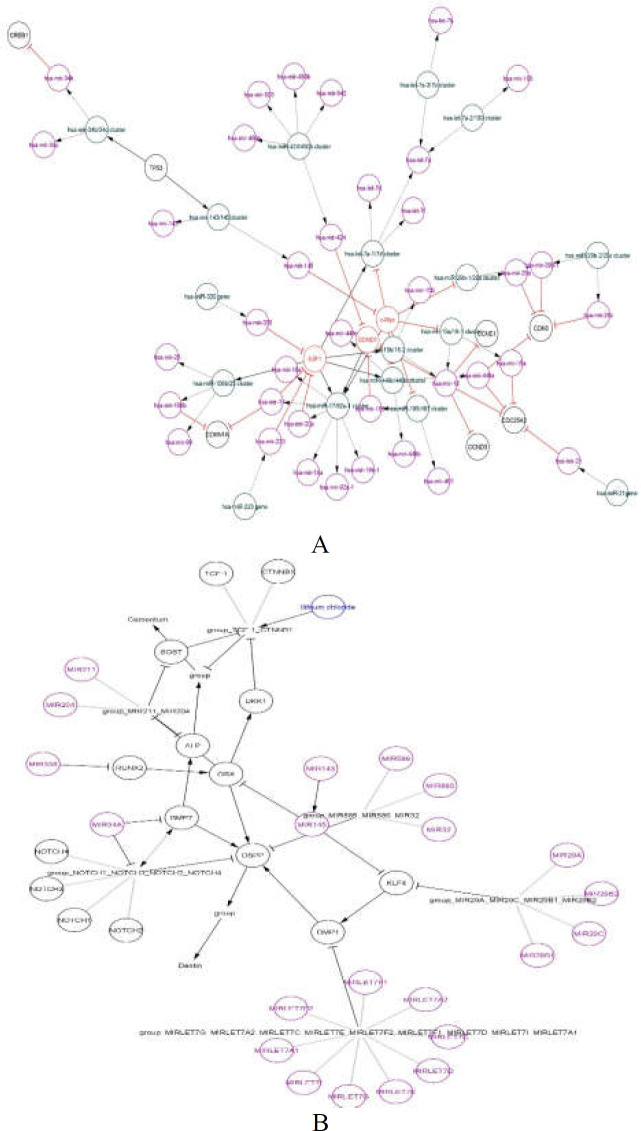
miR-21 (A) and miR-29 (B) target genes in regulatory pathways

The software Cytoscape showed the network analysis for two studied miRNAs and their target genes in regulatory pathways (Figure 6). We used the software to identify the cellular functions that were associated with changes mediated by ELF-EMFs exposure at the level of miRNA expression.

## Discussion

Cancer is a complicated diseases triggered by genetic and environmental (host-environmental) factors (3, 4). Aluminum, solvents, pesticides and exposure to ELF-EMF are listed as the potential environmental factors (1-4). As non-ionizing radiations, ELF-EMFs potentially influence enzyme processes involved in biological systems, and could be associated with human diseases because they can affect several biological functions, such as gene expression, regulation of cell fate, and cell differentiation (35-38). ELF-EMF can also modify the biophysical properties of cell membranes including their permeability to Ca2+ ions. Thus, several studies have investigated the effects of ELF-EMF on calcium homeostasis and cell differentiation (8, 9, 11, 13). Despite the fact that literature has explored the effects of ELF-EMF on cell proliferation, the results of different studies considerably vary as they have focused on experimental models and the applied fields of various characteristics (6, 7). A large number of studies have revealed the biological effects of ELF-EMFs on cancers (18-26). However, the underlying molecular mechanism of this influence remains unclear (6, 7). Recently, ELF-EMFs were shown to have no toxicity and adverse side effects in patients with advanced cancer (12, 15). The anti-tumor effect of ELF-EMFs is based on a mechanism that entails tumor cell apoptosis, promoting ROS production, and regulating anti-tumor immune system as well (11-16). 

Although ELF-EMFs have been long used in a wide range of therapeutic or diagnostic medical procedures, using them in cancer treatment is basically an emerging concept, of interest especially where conventional treatment options fail to serve the purpose they normally do (10-14). By the way of illustration, ELF-EMFs could be of great value to address untreatable tumors where the blood barrier is not penetrable for certain high molecular weight and/or hydrophilic anti-cancer drugs (15, 16). Moreover, ELF-EMFs will be of use in case other procedures (radiotherapy, chemotherapy, targeted antibodies, or other pharmacological treatments) fail to eliminate malignant tissues (21-24). Besides, a range of frequencies within the ELF-EMF spectrum could be used, in the presence or absence of signal modulation, to provide a therapeutic solution for particular tumors (10-14). This could be managed either by electricity (Tumor Treating Fields, electroporation), ELF-EMFs (hyperthermia, Radiofrequency electromagnetic fields treatment), chemotherapeutics or nanoparticles (6, 10, 16). Previous studies have so far addressed the question as to whether modulated ELF-EMFs at certain frequencies affect proliferation of human tumors (18-26). Yet, Tumor Treating Fields (TT Fields) are newly-introduced non-invasive treatment modalities with roots in electric fields (6, 7).

The present study investigated the diagnostic and prognostic values of miR-21 and miR-29 for gastric cancer once exposed to low frequency electromagnetic fields. According to the results of this study and their comparison with other similar studies (17-20), it was confirmed that ELF-EMFs could have anti-tumor effects on the expression of microRNAs in gastric cancer cell line. We also found that ELF-EMFs could up-regulate the expression levels of miR-21 and miR-29 when they are under-expressed in tumor cell lines. Furthermore, we tended to introduce these two miRNAs as potential biomarkers for gastric cancer diagnosis and prognosis. 

MicroRNAs (miRNAs) are among prominent mediators of host-environment interactions, i.e., hot spots in cancers (8-10). Literature has it that they are frequently over-expressed or under-expressed in human cancers (21-32). Thus, as is reported, any changes in miRNA expression can develop and encourage cancer progression (8, 9). There are several studies concerning miR-21 and miR-29 in gastric cancers (5, 21-32). Meanwhile, the same miR-21 and miR-29 could be potentially used as biomarkers for gastric cancer diagnosis (5, 21-31). Besides, a number of studies showed that the expression levels of miR-21 and miR-29 are sources of prognostic information for patients with gastric cancer independent of a comprehensive panel of other established clinical predictors (27, 29-31, 39-43). Shiotani et al. discovered that miR-21 could act as a fresh reliable marker of any rise in the risk of early gastric cancer after *Helicobacter pylori* eradication (39). Cui et al. found that there was a significantly higher level of miR-21 in gastric juice of the patients with gastric cancer than that of normal people (40). Li et al. also discovered that plasma levels of miR-21 were significantly higher in GC patients than in healthy controls (41). Wang et al. demonstrated the potential diagnostic and prognostic roles of miR-21 and miR-29 expressions in patients' tissues (27). Similarly, Moreira et al. reported that both miRNAs were aberrantly expressed (miR-21 was over-expressed and miR-29 was under-expressed) in the tumor tissue from miR-21 and miR-29 patients, while the expression profile of both miR-21 and miR-29 in healthy gastric antrum were 240 read counts and 444 read counts respectively (42). In another study, Zhao et al. showed that miR-29 was down-regulated in gastric cancer (16). Hwang et al. suggested that miR-29 expression could fall down in gastric cancer patients (30). Han et al. reported miR-29 expression had a significant part in gastric cancer cells by targeting ITGB1 pathway in a direct way (29). Gong et al. found that under-expression of miR-29 could serve as a tumor suppressor gene by accommodating critical oncogenic targets in gastric cancer (43). Accordingly, an oncogenic miRNA, miR-21 regulates a variety of downstream effectors involved with cancer. Overexpression of miR-21 is strongly connected with hematological and solid malignancies (8, 9). MiR-21 may be utilized as a diagnostic and prognostic biomarker for various types of cancer and as a potential therapeutic target (25-27). When over-expressed, miR-21 could change biological processes of gastric cancer cells such as proliferation, apoptosis, migration, and invasion probably through regulating RECK (a known tumor suppressor in gastric cancer) and other critical target genes (5, 21-27). Several studies reported miR-29 as a tumor suppressor gene, accommodating critical oncogenic targets, and indicated the potentials of using miR-29 as a biomarker of the gastric cancer diagnosis and its staging (27-31). 

Therefore, the present study focused on ELF-EMFs to be clinically applied to cancer treatment, and maintained that intermittent exposure to a 10 Hz ELF-EMFs could encourage changes in the cell viability and affect the expression of miRNAs in the gastric cancer cell line. Thus, we showed that miRNA regulation might play a pivotal role in boosting the biological impacts of a 10 Hz ELF-EMFs. The MTT assay was used to evaluate cell viability and it showed that exposure to 10 Hz ELF-EMFs could influence the proliferation of gastric cancer cells. In line with our findings, several studies demonstrated that exposure to ELF-EMFs could assess cell viability of tumor cells. For example, 10 Hz ELF-EMFs have significant effects on the cell growth, apoptosis, cell cycle and miRNA expression (18-20). To the best of our knowledge, previous studies did not consider the biological effect of ELF-EMF-induced changes in miRNA expression in the gastric cancer cell line. We also applied a Real-Time PCR to confirm the differential expression of miRNAs following 10 Hz ELF-EMFs exposure. We observed that miR-21 was the most highly up-regulated in gastric cancer cell line. MiR-21 was likely to work like an oncogene, targeting genes related to the cell cycle and apoptosis. In addition, up-regulation of miR-29 could suppress cell proliferation and induce senescence. Also, by overexpression in gastric cancer cell line, miR-29 could be a tumor suppressor and lead to the down-regulation of target pathways related to the cell arrest. Taken together, we demonstrated that intermittent exposure to a 10 Hz ELF-EMFs could alter the expression of miR-21 and miR-29 and could be regarded as anticancer activities. Our findings suggested that miR-21 and miR-29 could be used as biomarkers of ELF-EMF exposure, and might be key factors in biological effects of ELF-EMF. 

Finally, further research must be carried out to provide evidence for these claims. Obviously, further understanding of prognostic value of miRNAs could help clinical decision-making and develop miRNA-based target therapeutic treatments. Moreover, this study aimed to estimate the treatment of gastric cancer in Iran and account for discrepancies that probably contribute to designing and developing early cancer detection programs and conducting investigations on the risk factors that threaten Iranian population. Meanwhile, these studies lack a large sample size (as, patient and control groups) or an appropriate proportion of samples (as human or animal models). A reliable prediction of the survival requires large-scale samples with detailed clinical characteristics. Accordingly, ample research is needed to shed light on the networks regulated by the miRNAs which are involved in tumor growth and influenced by ELF-EMFs on one hand, and address the duration and the cycles of electromagnetic treatment on the other. 

In summary, we presented that intermittent exposure to a 10 Hz ELF-EMFs stimulated changes in miR-21 and miR-29 expressions in gastric cancer cell line. However, our findings suggested that miRNAs could be used as biomarkers for ELF-EMFs exposure, and the miRNAs regulation might be of great value in determining the biological effects of ELF-EMFs exposure. Such claims require further investigations. Our study revealed a novel mechanism behind the effect of ELF-EMFs on gastric cancer and provided a potentially useful adjunct therapy for diagnosis and treatment of this cancer.
